# Integrated morpho‐biochemical and transcriptome analyses reveal multidimensional response of upland cotton (*Gossypium hirsutum* L.) to low temperature stress during seedling establishment

**DOI:** 10.1002/pei3.10067

**Published:** 2021-11-20

**Authors:** Lakhvir Kaur Dhaliwal, Ritchel B. Gannaban, Avinash Shrestha, Junghyun Shim, Puneet Kaur Mangat, Joshua J. Singleton, Rosalyn B. Angeles‐Shim

**Affiliations:** ^1^ Department of Plant and Soil Science College of Agricultural Sciences and Natural Resources Texas Tech University Lubbock Texas USA; ^2^ Present address: Department of Nutritional Sciences College of Human Sciences Texas Tech University Lubbock Texas USA; ^3^ Present address: Olam International Limited Nasarawa Nigeria; ^4^ Present address: College of Agriculture, Food and Environment University of Kentucky Lexington Kentucky USA

**Keywords:** cold stress, electrolyte leakage, malondialdehyde, photosynthesis, reactive oxygen species

## Abstract

Cotton is a tropical/subtropical crop and is innately susceptible to cold. Using an approach that integrates morphological, biochemical, and transcriptome analyses, the study aimed to understand the molecular underpinnings of phenotypic adjustments in cotton seedlings under cold stress. Exposure of six cotton accessions to 15°C during the seedling stage significantly reduced chlorophyll content, stomatal conductance, plant height, and biomass, but increased malondialdehyde and proline production. Comparative transcriptome profiling of the cold‐sensitive accession SA 3781 grown under low and normal temperatures showed the upregulation of genes related to the production of reactive oxygen species (ROS) under cold stress. Despite a similar upregulation of genes encoding metabolites that can scavenge ROS and provide osmoprotection for the cell, the stressed plants still exhibited oxidative stress in terms of lipid peroxidation. This may be due in part to the upregulation of abscisic acid synthesis genes and downregulation of chlorophyll synthesis genes effecting lower stomatal conductance and chlorophyll contents, respectively. Additionally, stomatal closure which is required to avoid the cooling effect and dehydration under cold conditions may have contributed in reducing the net photosynthetic rates in plants exposed to low temperature. These findings provide an insight into the expression of key genes regulating the phenotypic changes observed in cotton in response to cold stress.

## 1 INTRODUCTION

Low temperature is a key environmental factor limiting the geographical distribution and productivity of crops that are adapted to tropical/subtropical climates (DeRidder & Crafts‐Brandner, 2008; Yadav, 2010). Depending on the onset and duration of cold stress, the severity of its impact to plant growth and development varies (Enders et al., 2019). Some of the earliest responses of a plant to cold exposure include modifications in cell membrane flexibility, changes in ionic fluxes, and reduced cytoplasmic streaming, accompanied by the excessive generation of highly reactive oxygen species (ROS). Together, these cellular disturbances set off a cascade of biochemical and physiological changes that become more pronounced and irreversible with prolonged stress exposure. The overproduction of ROS for instance, can result in the accumulation of cytotoxic by‐products due to ROS‐induced oxidation of biomolecules such as lipids, proteins, and nucleic acids. In its defense, the cell produces metabolites which function to scavenge excess ROS or repair intracellular oxidative damage (De los Reyes et al., 2018; Mehrotra et al., 2020; Ruelland & Zachowski, 2010). Failure to re‐establish the equilibrium between ROS production and scavenging disrupts cellular homeostasis, structure and function, severely compromising physiological processes including photosynthesis, carbohydrate metabolism, and photorespiration (Marocco et al., 2004). These impairments ultimately manifest in plants as poor growth, developmental delays, reduced biomass, leaf chlorosis and necrosis, and even death (Miedema, 1982; Yadav, 2010).

Upland cotton (*Gossypium hirsutum* L.) is a native of the tropics/subtropics and therefore has an intrinsic sensitivity to cold (Kittock et al., 1986; Krzyzanowski & Delouche, 2011; Speed et al., 1996). Despite its successful introduction and widespread cultivation in temperate environments, cotton flourishes best under long‐season cultivation in warm climates. Previous studies have determined the temperature ranges of 28–30 and 21–30°C to be the cardinal optimum for cotton during germination, and juvenile to adult vegetative phases, respectively (Lehman, 1925; Stanway, 1960). Conversely, 15°C has been established as the cardinal minimum or base temperature for the plant. Exposure to temperature equal or lower than 15°C any time during germination, seedling establishment, leaf area and canopy establishment, flowering and boll development, or maturation greatly impedes the overall growth and development of the cotton plant (Kittock et al., 1986; Krzyzanowski & Delouche, 2011; Speed et al., 1996). During germination for instance, imbibition at a critically low temperature of 10–12°C has been shown to significantly reduce germination rate, induce swelling of the hypocotyl base, and cause radicle injury, leading to embryonic root abortion followed by lateral root profusion (Bradow & Bauer, 2010; Shim et al., 2019). At the early seedling stage, cotton genotypes that are susceptible to cold have exhibited poor vigor characterized by stunting, seedling malformation, chlorosis, and taproot loss (Perry, 1980). During the reproductive stage, exposure to low temperatures has resulted in shedding of squares and abortion of flowers. At boll maturation, occurrence of cold snaps has been reported to significantly reduce cellulose production due to delayed fiber elongation and reduced cell wall thickening (Krzyzanowski & Delouche, 2011). Consequently, these physiological presentations have led to uneven crop stand and ultimately, to significant losses in yield and reduction in fiber quality (Kittock et al., 1986; Krzyzanowski & Delouche, 2011; Speed et al., 1996).

Efforts to elucidate the molecular underpinnings of low temperature responses in plants have identified various functional and regulatory genes that work synergistically to enhance tolerance to cold. The activation of regulatory genes such as transcription factors induces the expression of functional genes that directly or indirectly regulate downstream networks responsible for the observable physiological adjustments of plants under cold stress. One of the well‐studied pathways that promote cold tolerance relates to the regulation of the *C‐repeat binding factor/dehydration‐responsive element‐binding factor 1* (*CBF*/*DREB1*). Genes that encode the CBF/DREB1 proteins belong to a subclass of the *APETALA2*/*ethylene‐responsive* (*AP2*/*ERF*) superfamily of transcription factors. *CBF*/*DREB1* binds to the C‐repeat/dehydration‐responsive (CRT/DRE) cis‐elements in the promoter regions of a large number cold‐regulated genes to activate their expression (Jiao et al., 2016; Li et al., 2019; Mehrotra et al., 2020). In cotton, *GhDREB1L* has been reported to be induced in seedlings subjected to 4°C. *GhDREB1L* binds to the CRT/DRE‐like sequence in the promoter region of the *Late Embryogenesis Abundant D113* gene to enhance cold tolerance in cotton seedlings (Huang et al., 2007). In addition to *AP2*/*ERF*, regulons involving other superfamilies of transcription factors such as *WRKY*, *bHLH*, *bZIP*, *TCP*, *MYB*, and *C2H2* have also been identified to mediate tolerance to plants upon cold exposure (Chinnusamy et al., 2010; Mehrotra et al., 2020).

While various components of genetic pathways regulating cold stress responses are evolutionarily conserved in monocots and dicots, the effects of low temperature and the mechanistic responses to the stress is discrete and varies across and even within plant species. In cotton, independent studies have identified various morphological, physiological, and biochemical modifications in response to cold at different growth stages (Bradow & Bauer, 2010; DeRidder & Crafts‐Brandner, 2008; Krzyzanowski & Delouche, 2011; Shim et al., 2019). More recently, research aimed at providing a broader view of transcriptional changes under cold stress has led to the identification of genetic pathways and networks regulating cold stress responses (Cheng et al., 2020; Li et al., 2019). Despite these advances, information on the regulation of key genes or set of genes responsible for the observable morpho‐physiological and biochemical modifications in the cotton plant under cold stress is still scarce. Identification of candidate genes that can significantly contribute to the ability of the plant to tolerate low temperature stress will be critical in breeding efforts to improve this complex target trait in cotton. In this study, we used an integrative approach to provide a more comprehensive overview of the coordinated transcriptional, morphological, and biochemical changes occurring in cotton during seedling establishment in response to the established minimum cardinal temperature for the crop.

## 2 MATERIALS AND METHODS

### 2.1 Plant materials, growth conditions, and stress treatment

A set of upland cotton accessions composed of six obsolete varieties (SA 0033, SA 0718, SA 1156, SA 1232, SA 1766, and SA 3781) from the *Gossypium* Diversity Reference Set (GDRS; Hinze et al., 2015) were used to assess the effects of low temperature stress in cotton during the early seedling stage. The GDRS panel has been previously shown to have variable cold germination ability at 15°C (Shim et al., 2019).

Seeds of the experimental materials were directly sown and germinated in 72‐round plug trays filled with potting mix composed of 45–50% composted pine bark, vermiculite, Canadian sphagnum, peat moss, perlite and dolomitic limestone, and supplemented with basal NPK fertilizer (14–14–14 Osmocote classic). All materials were maintained in growth chambers set at 30°C. At the four true‐leaf stage, a set of 34 seedlings was transferred to a growth chamber set at 15°C, while another set was kept at 30°C as a control set‐up.

### 2.2 Morpho‐physiological and biochemical screening of cotton for cold stress responses at the early seedling stage

**FIGURE 1:**
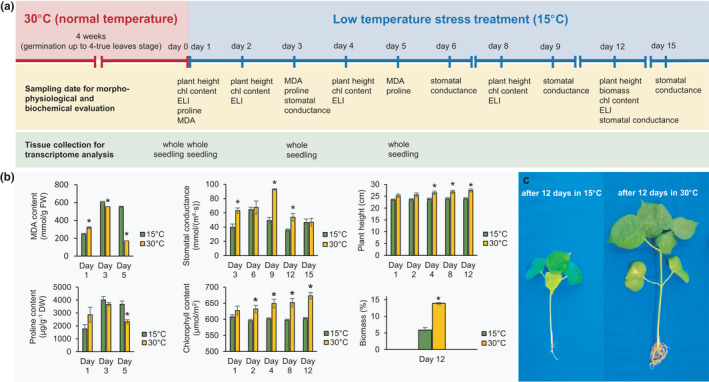
Experimental design and overall performance of SA 3781 under cold stress. (a) Schematic representation of the experimental design showing the different treatments and tissue sample collection for morphological, biochemical, physiological, and transcriptome analysis. (b) Physiological and biochemical screening of SA 3781 under cold and normal conditions. A total of six replications were used for plant height, chlorophyll content, biomass, and stomatal conductance, however, three replications were used for biochemical traits such as electrolyte leakage, malondialdehyde (MDA), and proline content. Statistical significance was determined by student's *t*‐test. Asterisks indicate significant differences at *p* ≤ 0.05. (c) Gross morphology of SA 3781 seedlings under cold and normal temperature. Bar = 1 cm

The effects of cold stress on cotton seedlings were assessed based on electrolyte leakage, lipid peroxidation, chlorophyll content, stomatal conductance, proline content, plant height, and biomass. Data on all the morpho‐physiological parameters were collected at different time‐points from seedlings maintained at 15 and 30°C (Figure 1A).

The extent of membrane damage caused by cold stress was assessed based on electrolyte leakage and lipid peroxidation assays using leaf tissues from both stressed and unstressed plants. Electrolyte leakage was measured from a total of 16 leaf discs punched from the edges of the four true leaves of a seedling. A total of three seedlings per genotype were used for ELI measurements. Briefly, the leaf discs were placed in a 50‐ml Falcon tube with 30 ml of deionized, sterile, distilled water. After shaking the tubes at 1500 rpm for 30 minutes, initial electrical conductivity of the water was measured using the Star A211 pH BT conductivity meter (Thermo Scientific). The samples were then boiled at 95°C for 1 h and cooled down to ≤50°C before measuring the final electrical conductivity of the water. Relative electrolyte leakage was calculated by dividing the measured conductivity before boiling with the total electrical conductivity after boiling (Cottee et al., 2010). The degree of lipid peroxidation in leaves was determined in terms of malondialdehyde (MDA) content (Tsikas, 2017). Approximately 75 mg of flash frozen leaf tissues collected from three different seedlings per genotype were collected in a 2 ml microcentrifuge tube containing 1.75 ml of 0.1% trichloroacetic acid (TCA). After vortexing the tube for 1 min and centrifugation at 10,000× g for 15 min, 357 μl of the supernatant was pipetted into a clean microcentrifuge tube with a punctured cap. The supernatant was mixed with 730 μl of 29% TCA and 750 μl of 0.5% w/v thiobarbaturic acid, and incubated in a water bath set at 95°C for 30 min. The tube was then immediately transferred on ice for 5 min to terminate the reaction. A 200 μl solution was pipetted on to an enzyme‐linked immunosorbent assay plate (Thermo Scientific) for absorbance reading at 532–600 nm using aplate reader (Thermo Scientific). Absorbance is read at 532 nm subsequent to subtraction of nonspecific absorption at 600 nm. MDA concentration was calculated using its extinction coefficient *ε* = 155 nM^−1^ cm^−1^ (Parry et al., 2014).

Chlorophyll content index (CCI) was determined from the first true leaf of six individual plants per genotype using the Apogee chlorophyll meter (Apogee Instruments). Since CCI only gives the relative indication of leaf chlorophyll content, the actual chlorophyll concentration (μmol/m^2^) was calculated using the conversion formula 84.3 + 98.6*(CCI)^0.505^. Cumulative changes in the CC values of each genotype were measured from the onset of stress up to day 12 of cold treatment. Stomatal conductance was measured from six plants per treatment using the SC‐1 leaf porometer (Decagon Devices).

To estimate proline content, approximately 100 mg of flash frozen tissue samples from three different seedlings per genotype were placed in a 2 ml tube with 400 μl of 0.5 w/v toluene. Samples were vortexed for 1 min, incubated at room temperature for 1 h, and centrifuged at 13,870 *g* for 10 min at 25°C. Approximately 100 μl of the solution was pipetted into a new tube containing 200 μl of glacial acetic acid and 200 μl of acid ninhydrin. The tubes were then punctured with a hypodermic needle and immediately incubated at 95°C for 1 h. The reaction was quenched in ice for 5 min before adding 1 ml of pure toluene onto the solution. Approximately 100 μl of the organic phase (top phase) of each sample was transferred into a 96‐well plate for absorbance reading at 520 nm. Proline concentration was determined from a standard curve and calculated on a fresh weight basis using the formula: [(μg proline/ml × ml toluene)/115.5 μg/μmole]/[(g sample)/5] = μmoles proline/g of fresh weight material.

The effects of cold stress on plant height (cm), which was measured from the base to the tip of the fully expanded first true leaf of six plants per genotype, were assessed based on cumulative changes in plant height from the onset of stress up to day 12 of cold treatment. Biomass was calculated using the formula: dry weight (g)/fresh weight (g) × 100% (Franks, 1997). Fresh and dry weights were based on measurements taken from six seedlings weighed in bulk after removing the root system and drying the aerial plant parts for 5 days in an oven set at 60°C.

### 2.3 Statistical analysis

Student's *t*‐test was conducted to establish differences in the values of all the parameters measured in response to the different temperature treatments. Statistical differences were determined at *p* ≤ 0.05.

### 2.4 Transcriptome analysis

Based on the morpho‐physiological and biochemical evaluation of the GDRS lines in response to low temperature stress, the upland cotton cultivar SA 3781 (cultivar Acala Royale) was selected for transcriptome analysis. Acala Royale is a high‐yielding, high‐quality cultivar released in the San Joaquin Valley in California in the 1990s. It has been previously identified to have robust germination ability (>80%) at 15°C (Shim et al., 2019) but poor vigor under the same low temperature at the early seedling stage based on the present study.

Seeds of Acala Royale were directly sown and germinated in 4″ × 4″ pots filled with potting mix and supplemented with basal NPK fertilizer as described previously. The materials were maintained in growth chambers set at 30°C. At the four true‐leaf stage, a set of 34 seedlings was transferred to a growth chamber set at 15°C, while another set was kept at 30°C as control.

After 0, 1, 3, and 5 days at 15°C or 30°C, the aerial part of three seedlings per treatment were collected, flash frozen in liquid nitrogen, and processed for RNA extraction using the RNEAsy kit (Qiagen) following the manufacturer's instruction. The integrity of the total RNA samples was evaluated using the NanoDrop™ One Microvolume UV‐Vis Spectrophotometer (ThermoFisher). All samples were outsourced to Novogene (Novogene Corporation Inc.) for library construction, quality control, and sequencing using the Illumina HiSeq platform.

Initial read quality (Q30, mapped reads, GC content) was evaluated using the FastQC application v0.11.2 (Anders & Huber, 2010). Pre‐processing of raw reads included the removal of adapter sequences and low‐quality reads. The clean sequence reads were then mapped to the available *G. hirsutum* (www.cottongen.org) and *Arabidopsis* genomes (https://www.arabidopsis.org/) using the HISAT2 algorithm. Transcript abundance and quantification of gene expression were estimated based on fragments per kilobase of transcript per million fragments mapped (FPKM; Trapnell et al., 2010). The ratio of FPKM values at 15 and 30°C was used to establish differential expression of the genes. Those with Log2 fold change of ≥2 or ≤−2 at *p* ≤ 0.05 was considered significantly differentially expressed genes (DEGs). Using this threshold, the regulation of DEGs related to each of the morpho‐biochemical traits observed in the study was examined. The Kyoto Encyclopedia of Genes and Genomes (KEGG) annotation information for gene IDs were used to identify the names and functions (Kanehisa & Goto, 2000) of genes relevant to the phenotypes observed in the plants under cold stress. For gene IDs lacking KEGG information, description for gene ontology (GO) components was considered as criterion to select the genes of interest. GO analysis to describe the features of DEGs at different time‐points under cold stress and normal condition were carried out using CottonFGD (https://cottonfgd.org/). GO analysis of DEGs was further classified under the broader GO terms of molecular function, biological function, and cellular components. Heat maps of the DEG were generated based on normalized expression values that show the relative expression of genes at two different conditions using the RStudio program (RStudio Team, 2020).

## 3 RESULTS

### 3.1 Morpho‐physiological and biochemical modifications in cotton plants under cold stress

The response of cotton seedlings to cold stress was evaluated based on physiological and biochemical adjustments, as well as observable morphological changes at variable time‐points within a 2‐week period. In general, the seedlings of all cotton genotypes performed poorly under cold than normal conditions. Seedlings subjected to 15°C recorded significantly lower chlorophyll content, stomatal conductance, percent change in plant height and biomass, and higher values for MDA and proline contents (Table 1; Tables S1–S7). The missing data points in Table 1 were due to the limited availability of SA 1232 and SA 1156 seeds during the conduct of the experiments which resulted in non‐replicated measurements. Electrolyte leakage from leaf tissues was not significantly different across genotypes and between treatments.

**TABLE 1:** Average values of different morphological, physiological, and biochemical parameters used to evaluate seedling vigor under cold stress (15°C) and normal temperature (30°C)

Genotype	Electrolyte leakage^b^ (µs/cm)		MDA content^a^ (mmol MDA/g FW)		Free proline content^a^ (µg/g DW)		Chlorophyll content^b,^ ^1^ (µmol/m^2^)		Stomatal conductance^c^ (mmol/(m² s))		Plant height^b,^ ^1^ (cm)		Biomass^b^ (g)	
	15°C	30°C	15°C	30°C	15°C	30°C	15°C	30°C	15°C	30°C	15°C	30°C	15°C	30°C
SA 0033	0.14	0.14	670.90	243.53*	357.30	95.10*	−0.35	8.73*	159.75	55.21	2.99	35.06*	5.67	12.92*
SA 1232	0.17	0.14	–	–	–	–	−3.40	7.13*	100.13	33.61*	3.43	19.79*	4.10	11.85*
SA 1766	0.13	0.14	375.10	309.50*	331.30	353.10	−3.59	6.89*	115.61	80.83	5.70	15.56	5.35	14.12*
SA 0718	0.13	0.13	737.10	597.60*	3771.10	144.80*	−4.39	8.05*	62.38	46.33	3.37	20.33*	6.41	11.56*
SA 3781	0.13	0.16	550.30	171.33*	3675.60	23200*	−1.94	7.38*	46.39	46.79	1.79	13.81*	5.87	13.47*
SA 1156	0.14	0.12	–	–	–	–	−4.17	7.43*	38.77	153.91*	3.43	9.49*	6.00	14.09*

Note

^a, b, c^ represent final measurements taken for each parameter, i.e., 5, 12, and 15 days of cold exposure, respectively.

Abbreviations: –, missing data points; MDA, malondialdehyde.

^1^ Values represent cumulative rate of change from day 1.

* Significant difference at *p* ≤ 0.05 between treatments.

Based on the reported good germination ability (Shim et al., 2019) but poor overall performance upon cold exposure, the cotton accession SA 3781 was selected for a more detailed analysis. SA 3781 seedlings subjected to 15°C exhibited lower proline and MDA contents at day 1 of cold exposure, although values for both parameters significantly increased by day 5 of cold stress. In contrast, stomatal conductance, chlorophyll content, plant height, and biomass remained consistently lower in plants maintained at 15°C than those at 30°C. Significant differences in the chlorophyll content and plant height of the stressed and non‐stressed plants were observed by days 2 and 4 of cold exposure, respectively. After 12 days of cold treatment, the stressed seedlings were remarkably smaller (Figure 1b,c) and therefore significantly lower in biomass compared to the unstressed plants.

### 3.2 Transcriptome sequencing and assembly

A summary of the sequencing assembly that provides the number of raw, clean, and mapped reads generated from SA 3781 at variable time‐points of exposure to 15 and 30°C is presented in Table 2. The number of raw reads ranged from 54,920,926 to 67,393,972. Upon the removal of adapter sequences and low‐quality reads, 53,649,794–66,896,302 clean reads were obtained. More than 90.42% of the clean reads mapped to the available *G. hirsutum* reference genome, with GC content ranging from 41.54% to 46.40%. The combined Q30 values of more than 94%, along with the high range of genomic coverage indicate the suitability of the RNA‐seq data for downstream analysis.

**TABLE 2:** Overview of RNA data obtained from RNAs extracted from aboveground tissue of SA 3781 under cold stress

Treatment		Raw reads	Clean reads	Mapped reads (%)	Error rate (%)	Q30 (%)	GC (%)
Temperature	Days						
30°C (control)	Day 0	54920246	54205882	94.04	0.02	95.05	45.39
15°C	Day 1	55729902	55034418	97.34	0.02	95.19	44.71
	Day 3	55409740	53649794	95.33	0.02	95.51	45.24
	Day 5	67393972	66896302	92.28	0.02	94.49	45.21
30°C	Day 1	58597888	57973990	93.35	0.02	95.41	45.67
	Day 3	58634688	57966832	90.42	0.02	95.42	46.40
	Day 5	59737330	57088196	92.27	0.02	94.50	41.54

### 3.3 Identification of cold‐responsive, DEGs

**FIGURE 2:**
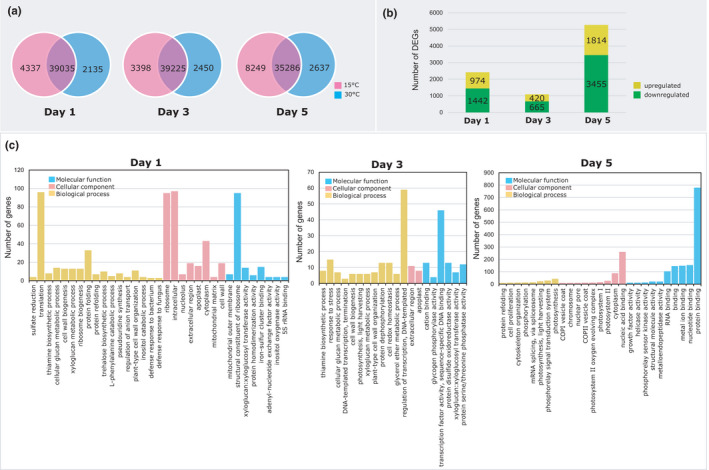
(a) Venn diagram depicting overall genes expressed under cold stress and normal conditions at different time‐points (1, 3, 5 days) after cold treatment. (b) Stacked bar graph represent the differentially expressed genes (DEGs) at three different time‐points (1, 3, 5 days) and depicts the upregulated and downregulated genes after cold treatment. Horizontal histograms represent GO classification of DEGs of cotton seedlings at 1, 3, 5 days of cold stress (c). A total of three different replications were used to conduct transcriptome analysis

A pairwise comparison of the total number of genes expressed in the aerial parts of SA 3781 under cold and normal conditions showed temporal variations in the overall transcript levels, with seedlings exposed to cold stress recording higher number of expressed genes (Figure 2a). From these, 2416, 1085, and 5269 genes were identified to be differentially expressed at 1, 3, and 5 days of cold treatment, respectively (Figure 2b). All identified DEGs were classified under 58 GO terms belonging to the three main categories of biological process, cellular components, and molecular functions (Figure 2c).

Analysis of the enriched GO terms showed the function‐based groupings of DEGs associated with the morphological and biochemical adjustments observed in plants after 1, 3, and 5 days of cold stress were identified. For instance, genes associated with chlorophyll biosynthesis were distributed in the most enriched GO terms related to “binding,” “nucleotide binding,” and “protein binding.” DEGs linked to chloroplast development, antenna complex, and photo‐ or non‐photochemical quenching were classified under photosynthesis‐related GO terms, such as “photosynthesis,” “photosystem I,” “photosystem II,” and “light harvesting.” Additionally, DEGs associated with stomatal conductance, MDA, and proline content grouped under the GO terms related to binding viz., “binding,” “nucleic acid binding,” “iron‐sulfur cluster binding,” “metal ion binding,” and “cation binding” (Figure 2c).

**FIGURE 3:**
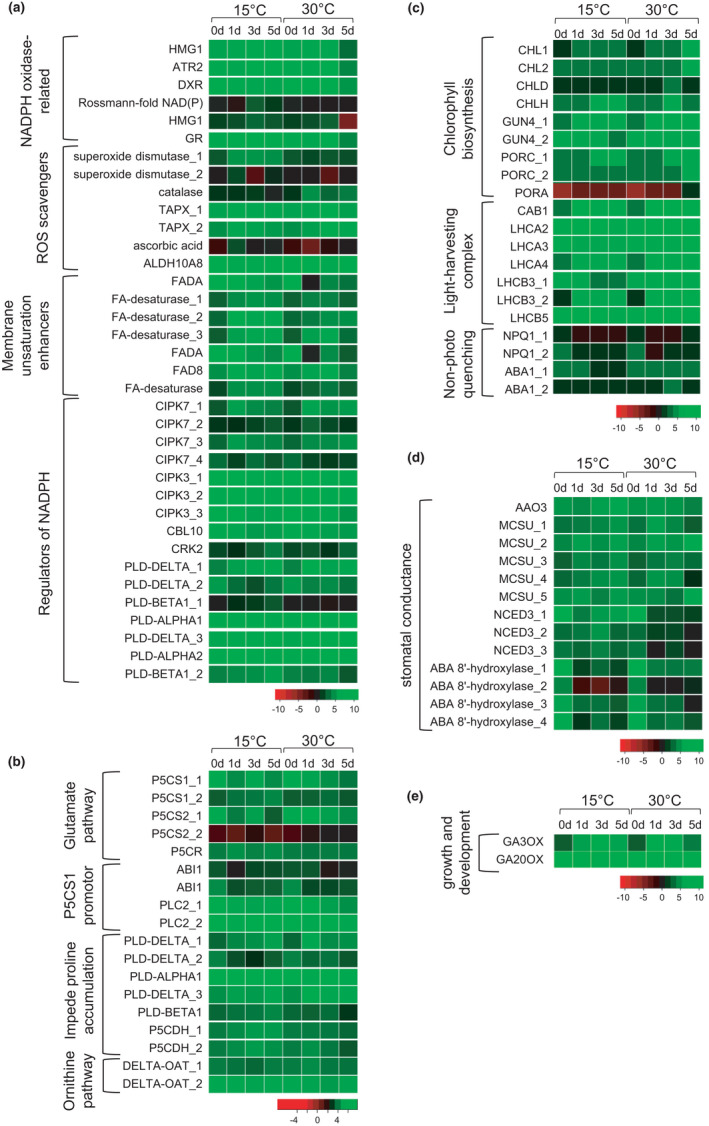
Heatmaps showing differential regulation of genes under cold stress and normal conditions. Illustrative representation of genes involved in (a) reactive oxygen species (ROS) production and scavenging along with regulation of nicotinamide adenine dinucleotide phosphate (NADPH), (b) proline biosynthesis via glutamate pathway, its promoters and inhibitors along with ornithine pathway, (c) chlorophyll biosynthesis, light harvesting complex, and non‐photo quenching genes, (d) stomatal conductance, (e) growth and development‐related genes in respective clusters. Normalized FPKM [Log2 (FPKM + 1)] values for each gene are from four different time‐points (0, 1, 3, and 5 days) under cold and normal conditions. Color gradient from red to green indicate low to high expression of genes

From the total of 8770 DEGs, 89 were determined to be involved in MDA and proline production, stomatal conductance, chlorophyll biosynthesis, and plant height. Of these, 38 were associated with MDA content as they relate to ROS production and scavenging, as well as cell membrane adjustments. In particular, genes encoding nicotinamide adenine dinucleotide phosphate (NADPH)‐oxidases including the positive regulators of these gene family were generally upregulated under cold stress (Figure 3a). Similarly, the expression of the genes encoding enzymatic (i.e., superoxide dismutase) and non‐enzymatic ROS scavengers (ascorbic acid), as well as various members of fatty acid desaturases, were upregulated in stressed cotton seedlings. As a defense mechanism, genes involved in the production of the known osmoprotectant proline via the glutamate and the ornithine pathways were also upregulated (Figure 3b; Table S8).

Conversely, the expression of genes associated with photosynthesis was generally downregulated in cotton seedlings exposed to cold stress. With the exception of *ABA1*, a gene involved in non‐photochemical quenching, genes that are related to chlorophyll biosynthesis and light harvesting complexes were downregulated in cold‐stressed plants (Figure 3c; Table S8).

Key genes that are involved in the synthesis of abscisic acid (ABA) namely *ABA‐aldehyde oxidase* (*AAO3*), *molybdenum cofactor sulfurase* (*MCSU*), and *9‐cis‐epoxycarotenoid dioxygenase* (*NCED3*) were upregulated, while genes encoding ABA‐8’‐hydroxylase, an enzyme that degrades ABA were downregulated under cold stress (Figure 3d; Table S8).

Lastly, variable expression of two GA biosynthetic genes i.e., *GA3 oxidase* and *GA20 oxidase* that are involved in plant growth and development were observed (Figure 3e; Table S8).

## 4 DISCUSSION

### 4.1 Early defense response of cotton seedlings to low temperature stress

To maintain thermal equilibrium under cold stress, plants have developed complex stress response mechanisms that are regulated at the molecular level and manifested as a series of physiological and biochemical adjustments (Cai et al., 2019; Iba, 2002). We examined the cold‐induced transcriptional changes in cotton seedlings to identify key genes or set of genes that are significantly contributing to the observed phenotypic modifications in the plants in response to low temperature stress.

One of the earliest responses of plants to cold stress is the loss of membrane flexibility. With prolonged cold exposure, membranes are forced into a rigid conformation that makes it prone to movement‐induced lesions (Sanchez et al., 2019; Simon, 1974; Willing & Leopold., 1983). Such membrane damage facilitates leakage of solutes out of the cell that mainly involves the efflux of monovalent potassium cations, along with its negatively charged counter ions (Bajji et al., 2002; Palta et al., 1977). In the present study, no considerable differences in electrolyte leakage were observed between the stressed and unstressed plants (Table 1). Kinetic studies on ion leakage from tomato pericarp discs have shown that increased penetrability in membranes develop very slowly in response to low temperature stress (Saltveit, 2002). In the case of cotton, it is possible that seedlings may require longer than 12 days of cold exposure before significant leakage of solutes out of the cell can be detected through ELI experiments.

Aside from electrolyte leakage, MDA has also been proven a good indicator of membrane damage. Plants overproduce ROS under extreme temperatures. While ROS are important signaling molecules that regulate normal plant growth in response to stress, their accumulation in the cell also causes oxidative stress on biomolecules such as nucleic acids, proteins, and lipids. Polyunsaturated lipids in membranes are damaged by ROS via the process of peroxidation. The extent of lipid peroxidation due to cold stress can be assessed by measuring MDA, the final product of oxidative lipid metabolism. In the present study, a significant increase in MDA content in the stressed seedlings may be indicative of a cold stress‐induced increase in cellular ROS.

The production of ROS in response to environmental stresses is the function of several classes of enzyme acting on various substrates. In the current study, a number of NADPH oxidase‐related genes which catalyzes the production of superoxide free radicles were upregulated at low temperature. Interestingly, the expression of the positive regulators of NADPH oxidase‐related genes such as the *CYSTEINE‐RICH RLK2 kinase* and *phospholipase D‐beta* were also upregulated, while negative regulators such as the *calcineurin B‐like*‐interacting protein kinases (*CIPK*s) were downregulated in stressed seedlings (Deng et al., 2013; Kimura et al., 2020; Mahajan & Tuteja, 2005; Park et al., 2004; You & Chan, 2015). Together, the observed patterns of regulation of these genes can contribute to ROS accumulation (Figure 3a; TableS8). In response to low temperature, the expression of fatty acid desaturase (FAD) genes was also upregulated. Lipid unsaturation of membranes is an adaptive response to avoid excessive solute leakage due to cold‐induced rigidity of membranes (Simon, 1974). While FA unsaturation lends more flexibility to membranes under cold stress, polyunsaturated FAs serve as substrates for ROS production.

To offset cellular imbalances, metabolites with protective functions against cold‐induced damages are produced in the cell. In the present study, the expression of genes for the enzymatic and nonenzymatic scavenging of ROS (i.e., superoxide dismutase, ascorbic acid), as well as genes related to the synthesis of the known osmoprotectant, proline (Chu et al., 1976; Kumar & Yadav, 2009; Muzammil et al., 2018; Tavakoli et al., 2016; Verbruggen & Hermans, 2008; Wang et al., 2020) were induced in the stressed plants. Proline biosynthesis in plants occurs via the glutamate and the ornithine pathways. The glutamate pathway involves a two‐step reaction starting with the generation of pyrroline‐5‐carboxylate synthase (P5C) from glutamate by *pyrroline‐5‐carboxylase synthase* (*P5CS*) gene followed by the conversion of P5C to proline by *pyrroline‐5‐carboxylase reductase* (*P5CR*) (Muzammil et al., 2018; Szabados & Savouré, 2010). With duplication events taking place in the complex eukaryotic genome, *P5CS* have two forms, that is, *P5CS1* and *P5CS2*. The *P5CS2* gene functions as a housekeeping gene for proline biosynthesis in the cytosol while the *P5CS1* regulates proline biosynthesis in the chloroplast in response to stress (Muzammil et al., 2018). In our study, *P5CS2* was downregulated under cold stress and is almost stationary at normal conditions (Figure 3b; Table S8). Conversely, *P5CS1*, along with *P5CR*, were upregulated in stressed seedlings which may account for the increased proline content in cotton seedlings exposed to cold temperature. Additionally, the downregulation of a *PLD* gene which suppresses the expression of *P5CS1* (Szabados & Savouré, 2010; Thiery et al., 2004), combined with the increased expression of the positive regulators *ABA insensitive 1* and *phosphoinositide phospholipase C2* of proline synthesis under stress (Knight et al., 1997; Strizhov et al., 1997) may have also contributed to the observed increase in proline content of the stressed plants.

Regulation of the *ornithine‐delta‐aminotransferase* (*delta‐OAT*) gene which mediates the biosynthesis of proline under the ornithine pathway was also observed in the cold‐stressed seedlings (Figure 3b; Table S8). Proline accumulation has been reported to be prevalent in seedlings under environmental stress (Armengaud et al., 2004). In the present study, two *delta‐OAT* genes (*delta‐OAT_1* and *delta‐OAT_2*) showed variable pattern of expression under cold and normal conditions, with *delta‐OAT_2*, exhibiting higher transcript levels under cold stress across all time‐points. The upregulation of this gene may have also contributed in the increase in proline content in cotton seedlings subjected to cold.

With increase in proline levels under cold stress, an upregulation in the expression of *pyrroline‐5‐caroxylase dehydrogenase* (*P5CDH*) gene was observed. *P5CDH* is involved in the catabolism of proline back to glutamate. The observed increase in the expression of this gene under cold stress may indicate a possible requirement to regulate the production of proline as a means to maintain osmotic balance in the cell.

### 4.2 Cold‐induced modifications in the photosynthetic apparatus

Photosynthesis is the primary source of energy in plants and is negatively regulated by low temperature stress (Banerjee & Roychoudhury, 2019). Considering the important roles of chlorophyll in harvesting and transducing radiation energy in antenna complexes, leaf chlorophyll content has been widely used to evaluate the photosynthetic capacity of crop plants (Eckhardt et al., 2004; Hajihashemi et al., 2018; Zhao et al., 2020). In the current study, the cumulative increase in the chlorophyll content of cotton plants at normal temperature was to ensure a healthy growth during the greening process (Figure 1). Upon exposure to 15°C, synthesis of chlorophyll content fell below normal levels in cotton leaves (Figure 1b). Comparative transcriptome analysis identified two genes encoding magnesium (Mg) chelatase and protochlorophyllide oxidoreductase (POR) which are involved in the biosynthesis of chlorophyll in leaves (Figure 3c). While both genes were upregulated in seedlings under normal temperature, their expression decreased under cold stress, potentially reducing chlorophyll biosynthesis. Magnesium chelatase is known to insert Mg^2+^ ions into protoporphyrin IX and this insertion has been reported as the first essential step for chlorophyll synthesis (Papenbrock & Grimm, 2001; Walker & Willows, 1997). The three different subunits of Mg chelatase are encoded by the *Mg chelatase subunit CHLI*, *CHLD*, and *CHLH* genes (Alberti et al., 2004; Jensen et al., 2000). Two of the three subunits namely *CHLI* and *CHLD* showed comparatively lower expression, whereas *CHLH* had a relatively higher expression under cold stress. The H subunit of Mg chelatase has been reported as an ABA receptor and signaling molecule (Shen et al., 2006; Wu et al., 2009). Previous study in *Arabidopsis thaliana* using surface plasmon resonance system demonstrated that ABA binds only to *CHLH*, but not to the *CHLI* and *CHLD* subunits of Mg chelatase (Du et al., 2012). A mutation in *CHLH* gene resulted in the insensitivity of stomata and seeds to the effects of ABA (Du et al., 2012). Given that ABA signaling is required for stomatal closure under cold stress, the potential role of *CHLH* as ABA receptor might explain its higher expression under cold stress.

The expression of Mg chelatase is positively regulated by a specific protein known as Genomes Uncoupled 4 (GUN4) (Larkin et al., 2003; Zhang et al., 2006). This regulator promotes Mg chelatase–substrate interactions by binding to the ChlH subunit of Mg chelatase (Larkin et al., 2003). In the present study, *GUN4* gene exhibited higher expression under normal conditions (Figure 3c). Conversely, the expression of *POR* genes which are responsible for the photoreduction of protochlorophyllide during chlorophyll biosynthesis was comparatively reduced in cotton seedlings exposed to cold. Similar findings were reported in rice seedlings subjected to cold and drought stress (Dalal & Tripathi, 2012; Zhao et al., 2020).

Chlorophyll biosynthesis is essential for the successful development of chloroplasts (Cortleven & Schmülling, 2015; Zhao et al., 2020). Within the chloroplasts are thylakoid membranes which provide the platform for light reactions during photosynthesis. Thylakoids contain three protein complexes viz., photosystem II (PSII), photosystem I (PSI), and the cytochrome b6‐f which participate in photosynthetic electron transport. In our study, low temperature stress reduced the expression of the light harvesting chlorophyll b‐binding protein (*Lhcb*) genes *Lhcb3*, *Lhcb5*, *Lhca3*, and *Lhca4*, as well as the *chlorophyll binding A*/*B* gene (*CAB1*). These genes are required for the production of antenna proteins that are peripherally attached to PSII and PSI. Reduction in the activity of light harvesting complexes triggers non‐photochemical quenching which is a protective mechanism of plants against excessive heat energy (Björkman & Demmig‐Adams, 1995; Govindjee, 2014). In our study, the increased expression of *NPQ_1* which is involved in non‐photochemical quenching might be a mechanism to avoid photoinhibition under cold stress.

Stomata are small openings on leaves that directly regulate the processes of photosynthesis and transpiration. Stomatal conductance, which measures the degree of stomatal opening has been widely used as an indicator of photosynthetic capacity and water status under stress (Ku et al., 2000; Thomas & Prasad, 2003). When stomatal pores open, CO_2_ becomes available to the plants triggering an increase in photosynthetic enzyme activity (Thomas & Prasad, 2003). Although enhanced stomatal conductance is directly proportional to internal CO_2_ concentration and photosynthetic rate, water loss through stomatal pores is also responsible for lowering leaf temperature (Ku et al., 2000). Plants exposed to low temperature thus tend to close their stomata in the expense of reduced photosynthetic capacity. In the present study, the reduced stomatal conductance of cotton seedlings exposed to 15°C (Figure 1b) might have contributed in reducing photosynthetic activity thereby decreasing plant height and biomass.

Abscisic acid is a phytohormone that directly regulates stomatal movement. Excessive production of ABA in response to environmental stress has been reported in a number of studies (Cutler & Krochko, 1999; Koornneef et al., 1998; Liotenberg et al., 1999; Taylor et al., 2000). In the present study, genes involved in ABA biosynthesis namely *AAO3*, *MCSU*, and *NCED3* were upregulated in chilled cotton seedlings (Figure 3d; Table S8). Interestingly, ABA‐8′‐hydroxylase which is involved in the degradation of ABA to phaseic acid showed decreased expression under cold stress compared to that of normal conditions (Figure 3d).

Given that plant growth and development are closely associated with photosynthetic capacity, the reduced chlorophyll content and stomatal conductance were expected to translate into decreased plant height and biomass under cold stress. In a previous study by Bai et al., 2016, reduction in GA biosynthetic genes has been identified to reduce cold‐induced reduction in cucumber growth and development. In the present study, the variable expression of *GA3 oxidase* in the stressed cotton seedling from 0 to 5 days of treatment, as well as the constant expression of *GA20 oxidase* in both stressed and unstressed plants are not in agreement with the observed reduction in plant height and biomass of cotton under cold conditions. Given that cold stress response is a very complex trait, the transcriptome result is indicative of other genes significantly contributing to both traits.

**FIGURE 4:**
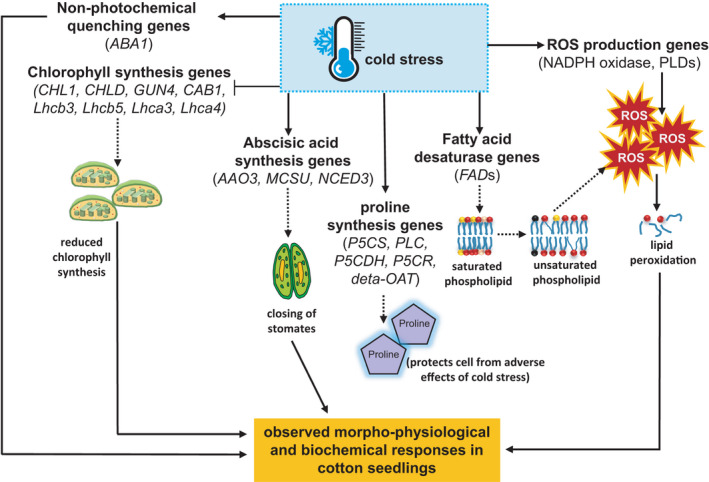
Proposed pathway depicting the cold stress responses of upland cotton during seedling establishment based on integrated morphological, biochemical, and transcriptome analyses. The overexpression of nicotinamide adenine dinucleotide phosphate (NADPH) oxidase‐related genes triggers excessive reactive oxygen species (ROS) accumulation, possibly leading to lipid peroxidation and thus malondialdehyde production. Overexpression of FAD genes enhances substrate availability for lipid peroxidation. Differential regulation of genes for proline biosynthesis via the glutamate and ornithine pathway facilitates proline production as a defense mechanism. The downregulation of chlorophyll biosynthetic genes and upregulation of abscisic acid synthesis genes controlling stomatal movement leads to the cold‐induced morphological changes in stressed seedlings. Broken arrow lines lead to products of gene expression

Based on our findings, a conceptual model integrating the transcriptome, cellular and morphological modifications in cotton subjected to low temperature stress during the early seedling establishment stage is presented in Figure 4. Cold perception by the plants triggers a multidimensional cascade of events that include the expression of genes toward the production of ROS and ABA, two signaling molecules that alerts the cells to the stress. In the cells' defense, genes for proline synthesis and fatty acid desaturation are expressed to provide osmoprotection and maintain membrane flexibility, respectively, under cold conditions. With prolonged cold stress however, the accumulation of ROS leads to oxidative stress on biomolecules including lipids while the overexpression of ABA synthesis genes compromises photosynthetic capacity by restricting stomatal opening. Because polyunsaturated FAs also serve as a substrate for ROS production, the upregulation of *FAD* genes is also indirectly contributing to the oxidative stress in cells. Under cold conditions, several genes that are necessary for the normal functioning of the photosynthetic apparatus are also downregulated that might have contributed to reduced plant growth and development.

## CONFLICT OF INTEREST

The authors declare no conflict of interest.

## Supporting information

Supplementary Material

## Data Availability

All the data are presented in figures, tables, and Supporting Information.
