# Supplementary material for: Integrated morpho‐biochemical and transcriptome analyses reveal multidimensional response of upland cotton (Gossypium hirsutum L.) to low temperature stress during seedling establishment
Source: Plant Environ Interact. 2021 Nov 20;2(6):290–302. doi: 10.1002/pei3.10067 (PMC10168043; doi:10.1002/pei3.10067)
Supplement: Supplementary file 1 — Supplementary Material [file PEI3-2-290-s001.docx]

Supplementary Tables

**Table S1.** Average electrolyte leakage in different genotypes across different time points under cold stress (15^o^C) and normal temperature (30^o^C).

| **Trait** | **Genotype** | **Day 1** | | **Day 2** | | **Day 4** | | **Day 8** | | **Day 12** | |
| --- | --- | --- | --- | --- | --- | --- | --- | --- | --- | --- | --- |
|  |  | **15°C** | **30°C** | **15°C** | **30°C** | **15°C** | **30°C** | **15°C** | **30°C** | **15°C** | **30°C** |
| Electrolyte leakage (µS/cm) | SA 0033 | 0.14 | 0.14 | 0.13 | 0.15 | 0.12 | 0.13 | 0.14 | 0.13 | 0.14 | 0.14 |
|  | SA 1232 | 0.18 | 0.16 | 0.23 | 0.16 | 0.15 | 0.15 | 0.15 | 0.14 | 0.17 | 0.14 |
|  | SA 1766 | 0.15 | 0.17 | 0.12 | 0.16^*^ | 0.16 | 0.14 | 0.14 | 0.13^*^ | 0.13 | 0.14 |
|  | SA 0718 | 0.15 | 0.16 | 0.13 | 0.15 | 0.14 | 0.13^*^ | 0.13 | 0.12 | 0.13 | 0.13 |
|  | SA 3781 | 0.14 | 0.17 | 0.14 | 0.21 | 0.14 | 0.13^*^ | 0.17 | 0.16 | 0.13 | 0.16 |
|  | SA 1156 | 0.18 | 0.16 | 0.15 | 0.19 | 0.16 | 0.14 | 0.15 | 0.15 | 0.14 | 0.12 |

* indicates significant differences at p ≤ 0.05

**Table S2.** Average malondialdehyde content in different genotypes across different time points under cold stress (15^o^C) and normal temperature (30^o^C).

| **Trait** | **Genotype** | **Day 1** | | **Day 3** | | **Day 5** | |
| --- | --- | --- | --- | --- | --- | --- | --- |
|  |  | **15°C** | **30°C** | **15°C** | **30°C** | **15°C** | **30°C** |
| MDA^a^ content (mmol MDA/g FW^b^) | SA 0033 | 48.50 | 472.37^*^ | 676.80 | 189.47^*^ | 670.90 | 243.53^*^ |
|  | SA 1766 | 299.80 | 485.37^*^ | 1042.10 | 449.00^*^ | 375.10 | 309.50^*^ |
|  | SA 0718 | 334.80 | 237.50^*^ | 464.60 | 314.07^*^ | 737.10 | 597.60^*^ |
|  | SA 3781 | 246.60 | 317.93^*^ | 609.30 | 555.43^*^ | 550.30 | 171.33^*^ |

^a^MDA = malondialdehyde

^b^FW = fresh weight

* indicates significant differences at p ≤ 0.05

**Table S3.** Average proline content in different genotypes across different time points under cold stress (15^o^C) and normal temperature (30^o^C).

| **Trait** | **Genotype** | **Day 1** | | **Day 3** | | **Day 5** | |
| --- | --- | --- | --- | --- | --- | --- | --- |
|  |  | **15°C** | **30°C** | **15°C** | **30°C** | **15°C** | **30°C** |
| Free proline content (µg/g DW^a^) | SA 0033 | 1986.70 | 1364.40 | 5208.90 | 1502.20^*^ | 357.30 | 95.10^*^ |
|  | SA 1766 | 1053.30 | 2862.20 | 8106.70 | 2831.10^*^ | 331.30 | 353.10 |
|  | SA 0718 | 1942.20 | 22089.00 | 7173.30 | 2675.60^*^ | 3771.10 | 144.80^*^ |
|  | SA 3781 | 1764.40 | 2862.20 | 4008.90 | 3675.60 | 3675.60 | 2320.00^*^ |

^a^DW = dry weight

* indicates significant differences at p ≤ 0.05

**Table S4.** Average stomatal conductance in different genotypes content across different time points under cold stress (15^o^C) and normal temperature (30^o^C).

| **Trait** | **Genotype** | **Day 3** | | **Day 6** | | **Day 9** | | **Day 12** | | **Day 15** | |
| --- | --- | --- | --- | --- | --- | --- | --- | --- | --- | --- | --- |
|  |  | **15°C** | **30°C** | **15°C** | **30°C** | **15°C** | **30°C** | **15°C** | **30°C** | **15°C** | **30°C** |
| Stomatal conductance (mmol/(m²·s)) | SA 0033 | 220.66 | 260.31 | 74.34 | 129.86^*^ | 99.34 | 174.20 | 104.26 | 118.29 | 159.75 | 55.21 |
|  | SA 1232 | 348.20 | 61.22^*^ | 116.90 | 99.61 | 113.30 | 405.74^*^ | 107.80 | 59.93^*^ | 100.13 | 33.61^*^ |
|  | SA 1766 | 242.49 | 205.76 | 189.80 | 234.33 | 209.61 | 200.33 | 138.26 | 104.58 | 115.61 | 80.83 |
|  | SA 0718 | 138.53 | 339.48^*^ | 96.02 | 245.47^*^ | 84.71 | 260.79^*^ | 67.17 | 91.00 | 62.38 | 46.33 |
|  | SA 3781 | 39.86 | 63.00^*^ | 64.34 | 67.47 | 49.10 | 92.85^*^ | 35.67 | 53.71^*^ | 46.39 | 46.79 |
|  | SA 1156 | 50.08 | 126.96^*^ | 51.30 | 222.99^*^ | 37.06 | 95.87^*^ | 58.19 | 57.74 | 38.77 | 153.91^*^ |

* indicates significant differences at p ≤ 0.05

**Table S5.** Average chlorophyll content in different genotypes across different time points under cold stress (15^o^C) and normal temperature (30^o^C).

| **Trait** | **Genotype** | **Day 1** | | **Day 2** | | **Day 4** | | **Day 8** | | **Day 12** | |
| --- | --- | --- | --- | --- | --- | --- | --- | --- | --- | --- | --- |
|  |  | **15°C** | **30°C** | **15°C** | **30°C** | **15°C** | **30°C** | **15°C** | **30°C** | **15°C** | **30°C** |
| Chlorophyll content (µmol/m^2^) | SA 0033 | 564.60 | 587.15 | 556.19 | 602.47^*^ | 554.50 | 615.99^*^ | 559.37 | 628.93^*^ | 562.45 | 644.66^*^ |
|  | SA 1232 | 550.71 | 559.71^*^ | 544.14 | 562.66^*^ | 551.92 | 582.70^*^ | 539.19 | 577.71^*^ | 531.97 | 598.33^*^ |
|  | SA 1766 | 596.07 | 595.99 | 588.30 | 601.99 | 584.76 | 606.53 | 584.59 | 624.09 | 578.10 | 636.32^*^ |
|  | SA 0718 | 593.51 | 589.42 | 580.24 | 588.51 | 588.35 | 609.67 | 583.75 | 614.59 | 565.13 | 640.99^*^ |
|  | SA 3781 | 607.96 | 627.32 | 595.80 | 631.95^*^ | 601.44 | 648.95^*^ | 597.25 | 651.94^*^ | 602.63 | 673.09^*^ |
|  | SA 1156 | 610.79 | 622.86 | 588.86 | 631.35^*^ | 595.11 | 656.74 | 591.35 | 647.97 | 590.21 | 667.00 |

* indicates significant differences at p ≤ 0.05

**Table S6.** Average plant height in different genotypes across different time points under cold stress (15^o^C) and normal temperature (30^o^C).

| **Trait** | **Genotype** | **Day 1** | | **Day 2** | | **Day 4** | | **Day 8** | | **Day 12** | |
| --- | --- | --- | --- | --- | --- | --- | --- | --- | --- | --- | --- |
|  |  | **15°C** | **30°C** | **15°C** | **30°C** | **15°C** | **30°C** | **15°C** | **30°C** | **15°C** | **30°C** |
| Plant height (cm) | SA 0033 | 29.40 | 26.00 | 29.60 | 26.40 | 29.80 | 28.00 | 30.10 | 29.40 | 30.30 | 33.20 |
|  | SA 1232 | 30.50 | 26.60 | 31.00 | 26.90 | 31.40 | 27.80 | 31.50 | 29.40 | 31.60 | 32.20 |
|  | SA 1766 | 25.10 | 22.50 | 25.80 | 23.20 | 26.10 | 23.90 | 26.40 | 24.60 | 26.50 | 26.90 |
|  | SA 0718 | 28.20 | 28.50 | 29.00 | 29.10 | 29.20 | 29.80 | 29.20 | 31.40 | 29.20 | 33.10 |
|  | SA 3781 | 23.40 | 25.20 | 23.50 | 25.60 | 23.70 | 26.40 | 23.90 | 26.80 | 23.90 | 26.60 |
|  | SA 1156 | 23.20 | 19.10 | 23.80 | 19.20 | 24.00 | 19.80 | 24.10 | 20.30 | 24.30 | 20.50 |

**Table S7.** Average biomass in different genotypes on day 12 under cold stress (15^o^C) and normal temperature (30^o^C).

| **Trait** | **Genotype** | **Day 12** | |
| --- | --- | --- | --- |
|  |  | **15°C** | **30°C** |
| Biomass (g) | SA 0033 | 5.67 | 12.92^*^ |
|  | SA 1232 | 4.10 | 11.85^*^ |
|  | SA 1766 | 5.35 | 14.12^*^ |
|  | SA 0718 | 6.41 | 11.56^*^ |
|  | SA 3781 | 5.87 | 13.47^*^ |
|  | SA 1156 | 6.00 | 14.09^*^ |

* indicates significant differences at p ≤ 0.05

**Table S8.** Normalized temporal expression of differentially expressed genes at cold stress (15^o^C) and normal temperature (30^o^C).

|  | **GeneID** | **Gene Name** | **15^o^C** | | | | **30^o^C** | | | |
| --- | --- | --- | --- | --- | --- | --- | --- | --- | --- | --- |
|  |  |  | **Control** | **Day 1** | **Day 3** | **Day 5** | **Control** | **Day 1** | **Day 3** | **Day 5** |
| NADPH oxidase related | Gh_A01G2017 | *HMG1* | 5.16 | 5.22 | 6.00 | 5.73 | 5.16 | 5.45 | 5.46 | 2.94 |
|  | Gh_A05G3999 | *ATR2* | 5.82 | 5.48 | 6.12 | 6.19 | 5.82 | 5.95 | 6.03 | 3.87 |
|  | Gh_A09G1555 | *DXR* | 6.03 | 5.96 | 5.49 | 5.24 | 6.03 | 6.01 | 6.57 | 4.45 |
|  | Gh_A11G2341 | *Rossmann-fold NAD(P)* | 0.49 | -0.80 | 2.30 | 1.31 | 0.49 | -0.16 | -0.09 | 0.00 |
|  | Gh_D04G2012 | *HMG1* | 1.43 | 1.96 | 2.70 | 2.20 | 1.43 | 1.98 | 2.49 | -4.08 |
|  | Gh_D11G1323 | *GR* | 5.99 | 6.62 | 6.56 | 6.47 | 5.99 | 5.42 | 6.13 | 3.90 |
| ROS scavengers | Gh_A07G0392 | *Superoxidase dismutase_1* | 2.03 | 4.40 | 4.58 | 4.33 | 2.03 | 1.78 | 2.20 | 1.98 |
|  | Gh_D05G0857 | *Superoxidase dismutase­_2* | 0.00 | 1.68 | -2.54 | 0.79 | 0.00 | 0.00 | -2.61 | 0.00 |
|  | Gh_A05G1539 | *catalase* | 1.20 | 1.17 | 1.31 | 0.52 | 1.20 | 4.24 | 2.77 | 3.57 |
|  | Gh_A06G0383 | *TAPX_1* | 5.10 | 5.80 | 5.48 | 5.41 | 5.10 | 5.33 | 6.55 | 4.80 |
|  | Gh_D06G0413 | *TAPX_2* | 4.35 | 5.28 | 5.10 | 4.71 | 4.35 | 4.87 | 6.36 | 5.27 |
|  | Gh_A13G1105 | *ascorbic acid* | -1.42 | 1.93 | -0.17 | 0.53 | -1.42 | -3.48 | -0.83 | 0.00 |
|  | Gh_A11G0380 | *ALDH10A8* | 9.30 | 7.43 | 7.45 | 7.14 | 9.30 | 9.09 | 8.17 | 7.01 |
| Membrane unsaturation enhancers | Gh_A01G0611 | *FADA* | 6.77 | 6.26 | 5.07 | 4.47 | 6.77 | 0.19 | 3.86 | 3.08 |
|  | Gh_A05G0325 | *FA-desaturase_1* | 2.52 | 4.18 | 4.49 | 4.35 | 2.52 | 3.70 | 3.71 | 2.55 |
|  | Gh_A07G1291 | *FA-desaturase_2* | 2.73 | 6.10 | 5.59 | 5.61 | 2.73 | 3.43 | 4.30 | 4.24 |
|  | Gh_A08G2217 | *FA-desaturase_3* | 2.39 | 4.95 | 5.45 | 5.41 | 2.39 | 4.89 | 5.49 | 2.82 |
|  | Gh_D01G0627 | *FADA* | 6.87 | 6.69 | 4.73 | 4.18 | 6.87 | 0.51 | 4.17 | 2.35 |
|  | Gh_D04G1274 | *FAD8* | 4.25 | 5.86 | 5.19 | 5.90 | 4.25 | 5.18 | 5.93 | 3.75 |
|  | Gh_D05G0430 | *FA-desaturase* | 1.76 | 4.37 | 4.30 | 4.21 | 1.76 | 2.52 | 3.28 | 2.39 |
| Regulators of NADPH | Gh_A02G0339 | *CIPK7_1* | 2.03 | 4.93 | 4.14 | 3.75 | 2.03 | 5.12 | 4.52 | 4.67 |
|  | Gh_A09G1937 | *CIPK7_2* | 1.11 | 0.94 | 1.54 | 2.02 | 1.11 | 2.44 | 1.75 | 1.03 |
|  | Gh_D02G0404 | *CIPK7_3* | 2.77 | 4.70 | 4.06 | 3.98 | 2.77 | 4.01 | 4.07 | 4.54 |
|  | Gh_D09G2147 | *CIPK7_4* | 2.81 | 1.47 | 2.84 | 2.19 | 2.81 | 1.60 | 1.28 | 1.69 |
|  | Gh_A05G0601 | *CIPK3_1* | 8.55 | 6.71 | 8.17 | 7.88 | 8.55 | 9.19 | 7.80 | 7.71 |
|  | Gh_D05G0732 | *CIPK3_2* | 8.11 | 6.45 | 7.55 | 7.47 | 8.11 | 8.74 | 7.77 | 7.45 |
|  | Gh_D07G1173 | *CIPK3_3* | 6.72 | 6.23 | 5.10 | 5.42 | 6.72 | 5.85 | 5.92 | 4.57 |
|  | Gh_D05G0440 | *CBL10* | 5.84 | 5.41 | 4.61 | 4.90 | 5.84 | 5.49 | 5.00 | 4.76 |
|  | Gh_A05G2721 | *CRK2* | 1.61 | 0.87 | 2.01 | 3.41 | 1.61 | 2.02 | 1.20 | 2.76 |
|  | Gh_A02G1674 | *PLDDELTA1* | 3.71 | 4.87 | 5.26 | 5.85 | 3.71 | 6.55 | 5.02 | 5.22 |
|  | Gh_A05G2168 | *PLDDELTA_2* | 4.66 | 2.91 | 2.00 | 3.25 | 4.66 | 3.70 | 4.07 | 3.13 |
|  | Gh_A08G1600 | *PLDBETA1_1* | 0.35 | 0.97 | 1.24 | 2.11 | 0.35 | 0.32 | -0.46 | 0.00 |
|  | Gh_A10G0662 | *PLDALPHA1* | 8.11 | 7.51 | 8.11 | 8.19 | 8.11 | 8.82 | 7.88 | 6.76 |
|  | Gh_D03G0048 | *PLDDELTA_3* | 5.23 | 5.80 | 5.70 | 6.32 | 5.23 | 7.05 | 6.57 | 6.43 |
|  | Gh_D10G0730 | *PLDALPHA2* | 7.23 | 6.53 | 7.27 | 7.50 | 7.23 | 7.29 | 6.69 | 5.78 |
|  | Gh_D11G0888 | *PLDBETA1_2* | 3.64 | 4.19 | 4.37 | 4.86 | 3.64 | 3.58 | 3.96 | 2.12 |
| Glutamate pathway | Gh_A04G1396 | *P5CS1_1* | 5.82 | 4.19 | 5.25 | 4.20 | 5.82 | 5.08 | 4.21 | 3.30 |
|  | Gh_D10G2321 | *P5CS1_2* | 2.33 | 3.19 | 3.20 | 3.97 | 2.33 | 2.40 | 2.99 | 2.12 |
|  | Gh_A01G1899 | *P5CS2_1* | 5.83 | 3.42 | 4.88 | 2.40 | 5.83 | 6.13 | 4.81 | 4.00 |
|  | Gh_D11G3311 | *P5CS2_2* | -2.08 | -2.98 | -0.82 | -2.83 | -2.08 | -0.69 | 0.00 | 0.00 |
|  | Gh_D07G0242 | *P5CR* | 3.40 | 4.26 | 3.76 | 4.17 | 3.40 | 3.36 | 3.58 | 3.21 |
| P5CS1 promoter | Gh_A13G1741 | *ABI1* | 2.10 | -0.08 | 1.68 | 1.81 | 2.10 | 2.05 | -0.45 | 0.43 |
|  | Gh_D06G0657 | *ABI1* | 4.11 | 1.89 | 2.30 | 2.41 | 4.11 | 1.77 | 1.63 | 2.01 |
|  | Gh_A06G1440 | *PLC2_1* | 6.23 | 5.31 | 5.11 | 5.24 | 6.23 | 6.40 | 4.86 | 4.25 |
|  | Gh_D06G2356 | *PLC2_2* | 6.76 | 5.86 | 6.22 | 6.12 | 6.76 | 7.35 | 6.08 | 5.55 |
| Impede proline accumulation | Gh_A02G1674 | *PLDDELTA_1* | 2.71 | 3.87 | 4.26 | 4.85 | 2.71 | 5.55 | 4.02 | 4.22 |
|  | Gh_A05G2168 | *PLDDELTA_2* | 3.66 | 1.91 | 1.00 | 2.25 | 3.66 | 2.70 | 3.07 | 2.13 |
|  | Gh_A10G0662 | *PLDALPHA1* | 7.11 | 6.51 | 7.11 | 7.19 | 7.11 | 7.82 | 6.88 | 5.76 |
|  | Gh_D03G0048 | *PLDDELTA_3* | 4.23 | 4.80 | 4.70 | 5.32 | 4.23 | 6.05 | 5.57 | 5.43 |
|  | Gh_D11G0888 | *PLDBETA1* | 2.64 | 3.19 | 3.37 | 3.86 | 2.64 | 2.58 | 2.96 | 1.12 |
|  | Gh_A03G0575 | *P5CDH_1* | 3.56 | 4.19 | 4.74 | 4.79 | 3.56 | 3.46 | 3.35 | 2.79 |
|  | Gh_D03G0856 | *P5CDH_1* | 3.48 | 4.01 | 4.70 | 4.31 | 3.48 | 4.30 | 3.27 | 2.08 |
| Ornithine pathway | Gh_A06G1211 | *DELTA-OAT_1* | 3.41 | 3.09 | 2.62 | 3.55 | 3.41 | 4.16 | 3.26 | 3.12 |
|  | Gh_D06G1509 | *DELTA-OAT_2* | 5.34 | 5.42 | 5.05 | 5.37 | 5.34 | 5.13 | 4.71 | 5.13 |

| Chlorophyll biosynthesis | Gh_A10G0282 | *CHLI1* | 5.61 | 7.09 | 7.36 | 7.88 | 5.61 | 6.75 | 7.42 | 8.80 |
| --- | --- | --- | --- | --- | --- | --- | --- | --- | --- | --- |
|  | Gh_D10G0283 | *CHLI2* | 6.76 | 7.24 | 7.31 | 7.44 | 6.76 | 6.97 | 7.32 | 9.37 |
|  | Gh_D08G2756 | *CHLD* | 5.84 | 6.15 | 6.12 | 6.23 | 5.84 | 5.71 | 6.58 | 5.47 |
|  | Gh_D09G1147 | *chlh* | 6.92 | 7.52 | 8.20 | 8.18 | 6.92 | 7.92 | 8.13 | 7.66 |
|  | Gh_A10G1223 | *GUN4_1* | 6.76 | 8.96 | 8.68 | 8.44 | 6.76 | 9.08 | 10.02 | 10.17 |
|  | Gh_D09G1997 | *GUN4_2* | 8.39 | 8.58 | 8.45 | 7.86 | 8.39 | 8.76 | 8.92 | 9.09 |
|  | Gh_A04G0903 | *PORC_1* | 7.05 | 7.87 | 8.29 | 8.51 | 7.05 | 7.94 | 8.20 | 8.84 |
|  | Gh_D04G1415 | *PORC_2* | 6.63 | 7.49 | 7.69 | 7.98 | 6.63 | 7.71 | 8.02 | 8.70 |
|  | Gh_A13G1234 | *PORA* | 0.55 | 2.59 | 2.10 | 1.52 | 0.55 | 1.59 | 2.34 | 5.67 |
| Light harvesting complex | Gh_A05G1261 | *CAB1* | 7.73 | 11.60 | 11.20 | 10.80 | 7.73 | 12.19 | 12.44 | 13.10 |
|  | Gh_D05G2361 | *LHCA2* | 10.27 | 11.76 | 11.74 | 11.39 | 10.27 | 11.84 | 12.37 | 13.03 |
|  | Gh_D11G1504 | *LHCA3* | 10.17 | 9.73 | 9.45 | 9.36 | 10.17 | 10.75 | 10.40 | 12.43 |
|  | Gh_D07G0661 | *LHCA4* | 7.80 | 9.38 | 8.22 | 8.32 | 7.80 | 10.73 | 10.64 | 11.27 |
|  | Gh_D05G3484 | *LHCB3_1* | 8.79 | 8.68 | 7.90 | 7.66 | 8.79 | 9.81 | 10.08 | 10.90 |
|  | Gh_D01G0531 | *LHCB3_2* | 5.41 | 9.23 | 9.15 | 8.50 | 5.41 | 10.43 | 10.70 | 11.72 |
|  | Gh_A05G2108 | *LHCB5* | 9.99 | 11.47 | 11.48 | 11.05 | 9.99 | 11.73 | 12.24 | 12.95 |
| Non-photo quenching | Gh_A11G2173 | *NPQ1_1* | 5.62 | 4.47 | 4.38 | 4.46 | 5.62 | 3.10 | 4.23 | 4.84 |
|  | Gh_D11G2473 | *NPQ1_2* | 7.05 | 5.46 | 5.09 | 5.56 | 7.05 | 4.48 | 5.16 | 5.89 |
|  | Gh_A11G1774 | *ABA1_1* | 7.55 | 6.83 | 5.87 | 5.73 | 7.55 | 6.47 | 7.19 | 6.37 |
|  | Gh_D11G3469 | *ABA1_2* | 6.19 | 5.78 | 5.19 | 5.29 | 6.19 | 5.77 | 6.46 | 5.81 |
| Stomatal conductance | Gh_D05G0771 | *AAO3* | 4.55 | 4.04 | 4.83 | 5.30 | 4.55 | 4.74 | 4.49 | 3.79 |
|  | Gh_A02G1820 | *MCSU_1* | 3.12 | 3.59 | 3.99 | 4.44 | 3.12 | 4.84 | 3.85 | 2.27 |
|  | Gh_A05G0049 | *MCSU_2* | 3.85 | 4.93 | 4.39 | 4.96 | 3.85 | 4.83 | 4.41 | 5.60 |
|  | Gh_A06G0127 | *MCSU_3* | 2.49 | 3.84 | 3.37 | 4.33 | 2.49 | 3.36 | 3.65 | 2.89 |
|  | Gh_D03G1678 | *MCSU_4* | 2.70 | 3.58 | 4.21 | 4.53 | 2.70 | 4.58 | 4.20 | 0.85 |
|  | Gh_D05G0102 | *MCSU_5* | 4.39 | 4.74 | 3.66 | 4.65 | 4.39 | 4.11 | 3.07 | 5.23 |
|  | Gh_A01G0280 | *NCED3_1* | 7.04 | 3.52 | 4.73 | 4.82 | 7.04 | 1.81 | 1.88 | 1.50 |
|  | Gh_A13G1311 | *NCED3_2* | 2.84 | 3.23 | 4.59 | 2.80 | 2.84 | 1.47 | 1.63 | 0.36 |
|  | Gh_D13G1614 | *NCED3_3* | 3.36 | 2.67 | 3.73 | 2.80 | 3.36 | -0.07 | 1.75 | 0.00 |
|  | Gh_A08G1344 | *ABA 8'-hydroxylase_1* | 6.24 | 1.58 | 2.66 | 1.68 | 6.24 | 3.58 | 3.30 | 3.40 |
|  | Gh_A12G1830 | *ABA 8'-hydroxylase_2* | 3.95 | -1.61 | -2.83 | -0.75 | 3.95 | 0.03 | 0.00 | 0.64 |
|  | Gh_D05G1939 | *ABA 8'-hydroxylase_3* | 5.10 | 2.89 | 3.09 | 2.23 | 5.10 | 3.04 | 2.90 | 0.38 |
|  | Gh_D08G1639 | *ABA 8'-hydroxylase_4* | 5.79 | 1.14 | 3.04 | 1.46 | 5.79 | 2.94 | 3.42 | 2.01 |
| growth and development | Gh_D06G2009 | *GA3OX* | 2.17 | 3.28 | 4.78 | 3.35 | 2.17 | 5.83 | 3.57 | 2.79 |
|  | Gh_A09G0044 | *GA20OX* | 5.44 | 4.71 | 4.91 | 4.91 | 5.44 | 4.66 | 4.55 | 4.96 |
